# Risk factors for left atrial thrombus in younger patients (aged < 65 years) with atrial fibrillation or atrial flutter: Data from the multicenter left atrial thrombus on transesophageal echocardiography (LATTEE) registry

**DOI:** 10.3389/fcvm.2022.973043

**Published:** 2022-10-12

**Authors:** Beata Uziȩbło-Życzkowska, Agnieszka Kapłon-Cieślicka, Monika Gawałko, Monika Budnik, Katarzyna Starzyk, Beata Wożakowska-Kapłon, Ludmiła Daniłowicz-Szymanowicz, Damian Kaufmann, Maciej Wójcik, Robert Błaszczyk, Jarosław Hiczkiewicz, Katarzyna Łojewska, Katarzyna Mizia-Stec, Maciej Wybraniec, Katarzyna Kosmalska, Marcin Fijałkowski, Anna Szymańska, Aleksandra Gos, Maciej Haberka, Michał Kucio, Błazej Michalski, Karolina Kupczyńska, Anna Tomaszuk-Kazberuk, Katarzyna Wilk-Śledziewska, Renata Wachnicka-Truty, Marek Koziński, Paweł Burchardt, Paweł Krzesiński

**Affiliations:** ^1^Department of Cardiology and Internal Diseases, Military Institute of Medicine, Warsaw, Poland; ^2^“Club 30”, Polish Cardiac Society, Katowice, Poland; ^3^1st Chair and Department of Cardiology, Medical University of Warsaw, Warsaw, Poland; ^4^West German Heart and Vascular Centre, Institute of Pharmacology, University Duisburg-Essen, Duisburg, Germany; ^5^Department of Cardiology, Maastricht University Medical Centre and Cardiovascular Research Institute Maastricht, Maastricht, Netherlands; ^6^Collegium Medicum, Jan Kochanowski University, Kielce, Poland; ^7^Department of Cardiology and Electrotherapy, Medical University of Gdańsk, Gdańsk, Poland; ^8^Department of Cardiology, Medical University of Lublin, Lublin, Poland; ^9^Collegium Medicum, University of Zielona Góra, Zielona Góra, Poland; ^10^Clinical Department of Cardiology, Nowa Sól Multidisciplinary Hospital, Nowa Sol, Poland; ^11^Department of Cardiology, School of Medicine in Katowice, Medical University of Silesia, Katowice, Poland; ^12^Members of the European Reference Network on Heart Diseases–ERN GUARD-HEART, Amsterdam, Netherlands; ^13^Department of Cardiology, St. Vincent Hospital, Gdynia, Poland; ^14^1st Department of Cardiology, Medical University of Gdańsk, Gdańsk, Poland; ^15^Department of Heart Diseases, Postgraduate Medical School, Warsaw, Poland; ^16^Department of Cardiology, School of Health Sciences, Medical University of Silesia, Katowice, Poland; ^17^Department of Cardiology, Medical University of Lodz, Lodz, Poland; ^18^Department of Cardiology, Medical University of Bialystok, Bialystok, Poland; ^19^Department of Cardiology and Internal Medicine, Medical University of Gdańsk, Gdynia, Poland; ^20^Department of Hypertension, Angiology, and Internal Medicine, Poznan University of Medical Sciences, Poznan, Poland

**Keywords:** atrial fibrillation, thromboembolic risk factors, young age group, left atrial thrombus, transesophageal echocardiography

## Abstract

**Background:**

Our aim was to assess the characteristics and to identify predictors of left atrial thrombus (LAT) in patients under age 65 with atrial fibrillation (AF) or atrial flutter (AFl).

**Methods:**

We conducted a subanalysis of a multicenter, prospective, observational study [the LATTEE registry]. Consecutive AF/AFl patients referred for cardioversion or ablation were enrolled.

**Results:**

Of the 3,109 patients included in the study, 1,276 were under age 65 (41%). Compared to non-LAT patients, those with LAT (*n* = 76) had higher CHA_2_DS_2_-VASc score (*p* < 0.001), more frequently had non-paroxysmal AF/AFl (*p* < 0.001), heart failure (*p* < 0.001), history of diabetes mellitus (*p* = 0.001), transient ischemic attack (*p* = 0.04), coronary artery disease (*p* = 0.02), and chronic kidney disease (*p* < 0.001). The LAT patients were also more often smokers (*p* = 0.004) and were more frequently treated with vitamin K antagonists (VKAs) (*p* < 0.001). Transthoracic echocardiography revealed a higher left atrial area (*p* < 0.001), lower left ventricular ejection fraction (LVEF) (*p* < 0.001), and lower value of LA appendage emptying volume in LAT than in non-LAT patients (*p* < 0.001). LVEF (OR 2.95; 95% CI: 1.32–6.59, *p* = 0.008), non-paroxysmal AF/AFl (OR 7.1; 95% CI: 2.05–24.63, *p* = 0.002) and treatment with VKAs (OR 4.92; 95% CI: 2.48–9.75, *p* < 0.001) were identified as independent predictors of LAT in younger patients.

**Conclusions:**

Our study, which focused on younger patients with AF/AFl, indicated substantial clinical and echocardiographic differences between participants with and without LAT. In the AF/AFl patients younger than age 65, the independent predictors of LAT included non-paroxysmal AF/AFl, lower LVEF, and treatment with VKAs.

## Introduction

Atrial fibrillation (AF) is a supraventricular arrhythmia primarily seen in the elderly (1, 2). The risk of thromboembolic complications in AF is closely related to age, as reflected in the strong prognostic power of this factor in the CHA_2_DS_2_-VASc scale and by clinical data showing an extremely high risk of stroke in patients over 80 years old (3, 4). A previous study showed that older patients with AF have a high risk of thromboembolic events, regardless of the other components of the CHA_2_DS_2_-VASc score (5). However, AF-related thromboembolic events also occur in young and middle-aged patients, even those with low CHA_2_DS_2_-VASc scores and without organic heart diseases (6, 7).

Only single reports have been made to describe predictors of left atrial thrombus (LAT) in younger AF patients. No consensus has yet been reached regarding whether thromboembolic risk in these subjects should be estimated based only on approved models for the general AF population (8). The aim of this study was to identify LAT predictors achievable before transesophageal echocardiography (TEE) in AF/AF flutter (AFl) patients under age 65.

## Materials and methods

### Patients

The analysis was carried out on a subgroup of patients from the left atrial thrombus on transesophageal echocardiography (LATTEE) registry (ClinicalTrials.gov Identifier: NCT03591627), which was a prospective, cross-sectional, observational, and multicenter study. Data were collected from consecutive AF or AFl patients who were enrolled at 13 cardiology centers. The reasons for admission were catheter ablation or cardioversion and TEE performed directly before the procedure. The methods of the study were previously described in detail (9, 10). Briefly, consecutive hospitalized patients diagnosed with AF/AFl who underwent TEE before direct current cardioversion or catheter ablation were included in the registry. The LATTEE registry was conducted in 13 Polish cardiology centers from November 2018 in the coordinating center and lasted 12 months or longer from the beginning of the study at each participating center (i.e., until the inclusion of at least 200 patients at each participating center; the last patient was enrolled in May 2020). Referral for TEE and the performance of other diagnostic tests depended on the routine practice of a particular center. All patients admitted for AF/AFl ablation were included in the study. Regarding non-emergency electrical cardioversion due to AF/AFL, four cardiology centers performed TEE on all patients, whereas nine centers performed TEE only on those patients suspected of not having received anticoagulant treatment in the previous 3 weeks.

Numerous data, including baseline demographic characteristics, AF type, medical history, concomitant diseases, laboratory and echocardiography test results, and current pharmacotherapy, were collected prospectively from all enrolled patients. Echocardiography examinations (transthoracic echocardiography [TTE] and TEE) were performed by certified echocardiographers. Echocardiographic data included left atrial area (LAa), left ventricular end-diastolic diameter (LVDd), left ventricular ejection fraction (LVEF), and left atrial appendage emptying velocity (LAAV). All echocardiographic measurements were conducted according to the current guidelines (11). The diagnostic criteria for heart failure (HF) with reduced EF (HFrEF), HF with mildly reduced EF (HFmrEF), and HF with preserved EF (HFpEF) were adopted for AF and AFl patients, as recommended in the European Society of Cardiology guidelines (3, 12). In this study, the presence of thrombus in the left atrial appendage (LAA) and spontaneous echocardiographic contrast (SEC) in both LA and LAA was evaluated. An LA thrombus was identified as a circular or irregular echodense mass in LA or LAA that was not part of the endocardium or pectinate muscles (13). The estimated glomerular filtration rate (eGFR) was calculated using the Cockcroft-Gault formula. Chronic kidney disease was defined as diagnosed kidney damage or a glomerular filtration rate (GFR) <60 mL/min/1.73 m^2^ for 3 months or more, irrespective of cause. The CHA_2_DS_2_-VASc score was calculated according to the current guidelines (3).

### Ethics approval statement

The study was conducted according to good clinical practice guidelines and the Declaration of Helsinki and was approved by the ethics committee (AKBE/113/2018). Data were entered anonymously into the registry database. The ethics committee waived the requirement to obtain informed consent from the patients.

### Statistical analysis

Statistical analyses were performed with IBM SPSS Statistics 25 (SPSS Inc., Chicago, IL, USA). Data were shown as a median and interquartile range or as the number of patients and percentages, where appropriate. The chi-square test was used to test the relationship between nominal variables and to confirm that the compared groups of people were equal. The Mann-Whitney U test was used to evaluate any statistically significant differences between the two independent groups. The Kruskal-Wallis test was used to assess whether statistically significant differences existed between more than two groups. If those differences were detected, an appropriate *post-hoc* test was used. The effect size and the effect of the quantitative factor on thrombus occurrence were measured using the Eta-squared values, which indicated which of the quantitative, statistically significant parameters were better at differentiating the compared groups of patients. The logistic regression analysis included the variables with the highest statistically significant differences (the *p*-value was considered significant when <0.05).

## Results

### Comparison of age-dependent study subgroups

In total, 3,109 AF or AFl patients were included in the study. Detailed data about the entire LATTEE registry group were included in previous manuscripts (9, 10).

As expected, the individual age groups of the AF and AFl patients differed in terms of many parameters. Younger patients were more often male, had the paroxysmal type of arrhythmia, and had significantly fewer comorbidities. These patients were also characterized by better heart function (LVEF and LAAV), higher body mass index (BMI), and better kidney function (measured by eGFR). Detailed data on the differences in individual age groups are shown in Table 1. LAT was detected in 76 (6.0%) patients aged 65 years or younger compared to 172 (9.4%) patients older than age 65 (*p* < 0.001).

**Table 1:** Characteristics of the study group according to age category.

**Variable**	**All study group**	**Age (years)**			***P*** **value**
		**<65**	**65–74**	**>74**	
**Type of AF/AFl**					
AF/AFl paroxysmal	1,280 (41)	652 (51)	475 (41)	151 (23)	<0.001
AF/AFl non-paroxysmal	1,819 (59)	621 (49)	679 (59)	516 (77)	<0.001
Time from AF/AFl diagnosis [years]	2 (1–5)	2 (1–5)	3 (1–5)	2 (1–6)	0.01
**Demographic data**					
**Sex**					
Female	1,145 (37)	309 (24)	479 (41)	355 (53)	<0.001
Male	1,963 (63)	967 (76)	681 (59)	313 (47)	
BMI [kg/m^2^]	29.3 (26.1–32.8)	29.5 (26–33)	30 (26–33)	28 (25–32)	<0.001
**Concomitant diseases**					
Heart failure	1,336 (43)	462 (36)	476 (41)	394 (59)	<0.001
HFrEF	476 (36)	202 (16)	159 (14)	114 (17)	0.12
HFmrEF	364 (27)	141 (11)	117 (10)	105 (16)	0.001
HFpEF	507 (38)	120 (9)	205 (18)	180 (27)	<0.001
Hypertension	2,366 (76)	844 (66)	944 (81)	573 (86)	<0.001
Diabetes mellitus	774 (25)	209 (16)	338 (29)	227 (34)	<0.001
Previous TIA	90 (28)	22 (2)	36 (3)	32 (5)	0.001
Previous stroke	235 (8)	70 (6)	82 (7)	82 (12)	<0.001
Coronary artery disease	905 (29)	211 (17)	409 (35)	283 (43)	<0.001
Chronic kidney disease	496 (16)	88 (7)	184 (16)	224 (34)	<0.001
Smoking	1,011 (33)	479 (39)	366 (33)	164 (26)	<0.001
Malignant tumor	106 (3)	19 (2)	52 (5)	35 (5)	<0.001
COPD	161 (5)	47 (4)	60 (5)	54 (8)	<0.001
CHA_2_DS_2_-VASc score [points]	3 (2–4)	2 (1–3)	3 (3–4)	5 (4–6)	<0.001
**Laboratory and echocardiography data**					
Hemoglobin [g/dL]	14.1 (13–15.2)	14.7 (13.6–15.6)	14 (12.9–15)	13.2 (12.1–14.3)	<0.001
eGFR [mL/min/1.73m^2^]	81.1 (61.5–104.7)	91.8 (74.57–112.95)	79.3 (62.4–97.8)	65.4 (49.35–83.85)	<0.001
LVEF [%]	55 (45–60)	55 (45–60)	55 (45.5–60)	50 (43–58)	<0.001
LA area [cm^2^]	26 (22–30)	25.3 (22–30)	26.95 (22.55–30.9)	26.7 (23–30.35)	<0.001
LAA emptying velocity [cm/s]	38.5 (26–53)	44 (31–63)	38 (26–50)	29 (21–40)	<0.001
**Oral anticoagulation therapy**					
VKA	500 (16)	174 (15)	212 (20)	113 (19)	0.01
Warfarin	217 (43)	77 (6)	94 (8)	45 (7)	0.13
Acenocumarol	283 (57)	97 (8)	118 (10)	68 (10)	0.049
**NOAC**	2,300 (74)	966 (85)	849 (80)	482 (81)	0.01
Rivaroxaban	1,070 (47)	466 (37)	399 (35)	204 (31)	0.03
Dabigatran	823 (36)	384 (30)	290 (25)	147 (22)	<0.001
Apixaban	407 (18)	116 (9)	160 (14)	131 (20)	<0.001

AF, atrial fibrillation; AFl atrial flutter; BMI, body mass index; COPD, chronic obstructive pulmonary disease; eGFR, estimated glomerular filtration rate; HFmrEF, heart failure with mildly reduced ejection fraction; HFpEF, heart failure with preserved ejection fraction; HFpEF, heart failure with reduced ejection fraction; LA, left atrial; LAA, left atrial appendage; LVEF, left ventricular ejection fraction; NOAC, Non-vitamin K antagonist oral anticoagulants; VKA, vitamin K anticoagulants.

Data are presented as median (interquartile range, as equal to the difference between upper and lower quartiles, IQR) and n (%).

### Comparison between younger patients with and without LAT

A subgroup of 1,276 patients younger than age 65 was subjected to further detailed analysis. The patients aged <65 years with LAT and non-LAT were comparable in terms of general parameters, such as sex (*p* = 0.68) or BMI (*p* = 0.51). Non-paroxysmal AF/AFl and comorbidities were significantly more frequent in the LAT subjects. Significant differences were also found in the echocardiographic parameters between the subgroups, as LAT patients had a larger left atrium size (LAa, *p* < 0.001), lower LVEF (*p* < 0.001), and lower LAAV (*p* < 0.001) (Table 2). Supplementary Table S1 shows that effect size measures for quantitative variables differentiated the LAT group from the non-LAT group.

**Table 2:** Detailed characteristic of patients aged < 65 years according to the presence of a left atrial thrombus.

**Variable**	**Patients**<**65 years**		***P*** **value**
	**LAT(-)**	**LAT(+)**	
**Type of AF/AFl**			
AF/AFl paroxysmal	640 (54)	12 (16)	<0.001
AF/AFl non-paroxysmal	556 (47)	65 (84)	<0.001
Time from AF/AFl diagnosis [years]	2 (1–5)	2(1–5)	0.46
**Demography data**			
**Sex**			
Female	289 (24)	20 (26)	0.68
Male	910 (76)	57 (74)	
BMI [kg/m^2^]	29 (26–33)	29 (26–32)	0.51
**Concomitant diseases**			
**Heart failure**	408 (34)	54 (70)	<0.001
HFrEF	165 (14)	37 (48)	<0.001
HFmrEF	125 (11)	16 (21)	0.01
HFpEF	119 (10)	1 (1)	0.008
Hypertension	788 (66)	56 (73)	0.26
Diabetes mellitus	185 (15)	24 (31)	0.001
Previous transient ischemic attack	18 (2)	4 (5)	0.04
Previous stroke	63 (5)	7 (9)	0.19
Coronary artery disease	190 (16)	21 (27)	0.02
Chronic kidney disease	74 (6)	14 (18)	0.001
Smoking	438 (38)	41 (55)	0.004
Malignant tumor	18 (2)	1 (1)	1
COPD	43 (4)	4 (5)	0.52
CHA_2_DS_2_VASc score [points]	2 (1–2)	3 (2–3)	< 0.001 eta^2^ = 0.03
**Laboratory and echocardiography data**			
Hemoglobin [g/dL]	14.7 (13.6–15.6)	14.6 (13.2 – 15.8)	0.50 eta^2^ = 0.002
eGFR [mL/min/1.73 m^2^]	92.1 (74.7–113)	89.1 (66.55–111.95)	0.23
LVEF [%]	55 (45–60)	38.4 (28–50)	< 0.001 eta^2^ = 0.05
LA area [cm^2^]	25 (21.5–29.5)	30 (27–35)	< 0.001 eta^2^ = 0.02
LAA emptying velocity [cm/s]	46 (34–65)	20 (16–27)	< 0.001 eta^2^ = 0.09
**Oral anticoagulation therapy**			
**VKA**	148 (14)	26 (44)	< 0.001
Warfarin	67 (6)	10 (13)	0.02
Acenocumarol	81 (7)	16 (21)	< 0.001
**NOAC**	933 (86)	33 (56)	< 0.001
Rivaroxaban	452 (38)	14 (18)	< 0.001
Dabigatran	371 (31)	13 (17)	0.01
Apixaban	110 (9)	6 (8)	0.84

Abbreviations as in Table 1.

Data are presented as median (interquartile range, as equal to the difference between upper and lower quartiles, IQR) and n (%).

### Predictors of LAT in patients with AF/AFl under 65 years of age

We next identified the LAT predictors that were achievable without performing the TEE. Our multivariate regression analysis included the following variables (which were statistically significant in univariable logistic regression): AF/AFl-non-paroxysmal, HF, diabetes mellitus (DM), coronary artery disease, smoking, treatment with vitamin K antagonists (VKAs), CHA_2_DS_2_-VASc score, LVEF, LAa, and chronic kidney disease (CKD). The independent LAT predictors (odds ratio; 95% confidence interval [CI]) were treatment with VKAs (4.92; 2.48–9.75; *p* < 0.001), LVEF value (2.95; 1.32–6.59; *p* = 0.008), and non-paroxysmal AF/AFl (7.1; 2.05–24.63; *p* = 0.002) (Table 3).

**Table 3:** Results of univariable and multivariable logistic regression analysis – predictors of left atrial thrombus.

**Variable**	**Odds ratio; 95% CI**	
	**Univariable logistic regression analysis**	**Multivariable logistic regression analysis**
AF/AFl non–paroxysmal	6.23; 3.33–11.66 (*p* < 0,001)	7.1; 2.05–24.63 (*p* = 0.002)
HF	4.53; 2.74–7.48 (*p* < 0.001)	1.05; 0.4–2.71 (*p* = 0.92)
Diabetes mellitus	2.48; 1.49–4.12 (*p* < 0.001)	1.78; 0.78–4.04 (*p* = 0.17)
Coronary artery disease	1.99; 1.18–3.37 (*p* = 0.01)	0.65; 0.27–1.58 (*p* = 0.35)
Smoking	2; 1.25–3.22 (*p* = 0.003)	1.12; 0.56–2.23 (*p* = 0.75)
VKA	4.97; 2.89–8.54 (*p* < 0.001)	4.92; 2.48–9.75 (*p* < 0.001)
CHA_2_DS_2_VASc	3.27; 2.05–5.21 (*p* < 0.001)	1.81; 0.73–4.53 (*p* = 0.2)
LVEF	6.64; 4.03–10.94 (*p* < 0.001)	2.95; 1.32–6.59 (*p* = 0.008)
LA area	3.96; 2.3–6.82 (*p* < 0.001)	1.57; 0.76–3.21 (*p* = 0.22)
Chronic kidney disease	3.38; 1.8–6.31 (*p* < 0.001)	0.92; 0.36–2.4 (*p* = 0.87)

Abbreviations as in Tables 1, 2.

The cut-off values for continuous variables were their medians: CHA_2_DS_2_VASc, 3 points; LVEF, 38.4%; LA area, 30 cm^2^.

## Discussion

In the group of patients with AF or AFl and younger than age 65, we successfully identified clinical (non-paroxysmal AF/AFl), echocardiographic (LVEF), and therapeutic (treatment with VKAs) LAT predictors. The multivariable logistic regression revealed that other well-established stroke risk factors, such as diabetes mellitus, coronary artery disease, and even the CHA_2_DS_2_VASc score, were not independent of these variables.

As expected, the LAT prevalence was less frequent in younger patients with AF and AFl than in older patients with AF/AFl, in agreement with previous studies (8, 14). Subjects under age 65 who were burdened with cardiovascular comorbidities, such as HF, DM, previous transient ischemic attack (TIA), coronary artery disease, and CKD, were more likely to have LAT. Some comorbidities (HF, DM, and TIA) are components of the CHA_2_DS_2_-VASc scale; therefore, their role in increasing thromboembolic risk has been proven. Likewise, CKD has recently been reported as a significant risk factor for thromboembolism in AF patients (15–17). Kapłon-Cieślicka et al. (15) demonstrated that a new CHA_2_DS_2_VASc-RAF score (where R stands for renal dysfunction and AF for non-paroxysmal AF) was better than the CHADS and CHA_2_DS_2_VASc scales used to predict LAT in patients with AF.

In our study, younger patients with AF/AFl smoked more often than older ones from the whole LATTEE group. The univariate analysis identified smoking as a risk factor for thrombus in the left atrium or left atrial appendage, but this result was not confirmed by multivariate analysis. Nevertheless, the association between smoking and the risk of AF occurrence or thromboembolism is inconclusive. Chamberlain et al. (18) showed a more than twofold increased risk of AF attributed to smoking, whereas current smoking was not a significant AF predictor in the Framingham risk score (19). Inao et al. (20) revealed that current smokers had reduced LAAV and more often had dense left atrial spontaneous echo contrast, indicating an impact of smoking on thromboembolic risk in patients with AF. Other authors also found that smoking was associated with a higher thromboembolic risk in AF patients, even after adjustment for well-recognized risk factors used in stroke risk stratification schemes (21). In the present study, among the many factors differentiating subgroups of young patients with AF/AFl with and without LAT, we found only three parameters that were independent risk factors for LAT: non-paroxysmal AFl, LVEF, and treatment with VKAs.

An increased risk of thromboembolism has been previously shown due to non-paroxysmal AF/AFl (15, 22, 23). An extensive systematic review and meta-analysis, including 12 studies containing 99,996 patients with AF, showed an unadjusted risk ratio of 1.355 (*p* < 0.001) for thromboembolism in non-paroxysmal AF vs. paroxysmal AF. Moreover, non-paroxysmal AF patients had a higher risk of all-cause mortality (1. 217; *p* < 0.001) (24). Lin et al. (25) retrospectively analyzed a group similar to ours (mean age 52.10 ± 9.64 years) in terms of the clinical, laboratory, and echocardiographic data of 705 patients with non-valvular AF and low risk of stroke (CHA_2_DS_2_-VASc score of 0 or 1) and found an independent association between non-paroxysmal AF and LAT or spontaneous echocardiographic contrast in those patients (OR = 3.766, 95% CI: 1.282–11.061, *p* = 0.016). In that study, approximately 80% of patients with LAT/SEC had non-paroxysmal AF, which is comparable to our result of 84% in the present study.

Many recent studies have confirmed the advantage of non-vitamin K antagonist oral anticoagulants (NOACs) over VKAs in reducing thromboembolic risk (26, 27). These results are reflected in the current guidelines, in which NOACs are explicitly recommended as a choice over VKAs to treat patients with AF. In our previous study, the LAT prevalence in the group of AF patients with IIa class anticoagulation recommendations was higher in the AF patients treated with VKAs than with NOACs (8.4 vs. 3.4%, *p* = 0.010). Our multivariate logistic regression revealed a threefold higher LAT risk after treatment with VKAs than with NOACs (28). However, our study was observational and we were unable to estimate a time in therapeutic range (TTR) for our participants. We have only the INR result performed at admission for 482 of the 500 patients treated with VKAs, and the mean value was 2.5 ± 1.1. The lower relative efficacy of VKAs in our observational registry may be explained by the lower time in the therapeutic range in real-world patients compared to the time in the therapeutic range achieved in patients included in clinical trials. This could suggest an even greater benefit of NOACs compared to VKAs in the real-world AF population.

Our finding of a negative prognostic value for left ventricular systolic dysfunction is in agreement with other reports confirming decreased LVEF as a powerful and independent predictor of LAT formation in AF patients (24). Melduni et al. (29) reported an LVEF value of ≤ 40% as significantly increasing the probability of LAT. Likewise, in our previous study, which consisted of 768 AF patients (mean age, 63 years), LVEF was also one of the independent LAT predictors (30). Recently, a large Chinese study (36,007 NVAF inpatients from 602 hospitals) identified a lower LVEF as an important predictor of LAT (31). The authors demonstrated that a higher LVEF (per 5%, OR 0.95, 95% CI 0.92–0.97, *P* = 0.017) indicated a reduced prevalence of LAT.

## Conclusions

Our results suggest that the pathogenesis of LAT formation in AF patients is complex and that other components of the CHA_2_DS_2_-VASc scale should also be considered. In our study, the independent predictors of LAT in patients younger than 65 with AF/AFl were: non-paroxysmal AF or AFl, LVEF, and treatment with vitamin K antagonists.

## Limitations

The registry-based character of this multicenter study imposes some limitations on our findings. First, the echocardiographic examinations were performed by numerous physicians (at least 1 at each study site). This fact might have had some impact on the obtained results, especially regarding possible measurement bias in assessing the presence of LAT. However, at each center, all examinations were performed by experienced echocardiographers, and if a thrombus was suspected, all images were reviewed by a second experienced echocardiographer trained in TEE. A second limitation is that we evaluated the presence of LAT, assuming it to be a surrogate of a high risk of thromboembolism. Finally, no follow-up was conducted for clinical events; therefore, we could not verify the value of our model in predicting long-term patient prognosis.

## Data availability statement

The original contributions presented in the study are included in the article/Supplementary material, further inquiries can be directed to the corresponding author.

## Ethics statement

The studies involving human participants were reviewed and approved by Ethics Committee of Medical University of Warsaw. Written informed consent for participation was not required for this study in accordance with the national legislation and the institutional requirements.

## Author contributions

BU-Ż, AK-C, MG, and PK: conceptualization and methodology. BU-Ż: formal analysis and writing–original draft preparation. BU-Ż and PK: resources. PK, AK-C, and MKo: writing–review and editing. BU-Ż, PK, and AK-C: visualization. PK, AK-C, and BW-K: supervision. All authors contributed to investigation, data collection and manuscript revision, read, and approved the submitted version.

## Conflict of interest

Author AK-C honoraria for lectures from Bayer, Boehringer Ingelheim, Pfizer, outside the submitted work. The remaining authors declare that the research was conducted in the absence of any commercial or financial relationships that could be construed as a potential conflict of interest.

## Publisher's note

All claims expressed in this article are solely those of the authors and do not necessarily represent those of their affiliated organizations, or those of the publisher, the editors and the reviewers. Any product that may be evaluated in this article, or claim that may be made by its manufacturer, is not guaranteed or endorsed by the publisher.

## Abbreviations

AF: atrial fibrillation
AFl: atrial flutter
BMI: body mass index
CKD: chronic kidney disease
DM: diabetes mellitus
eGFR: estimated glomerular filtration rate
ESC: European Society of Cardiology
HF: heart failure
HFmrEF: heart failure with mildly reduced ejection fraction
HFpEF: heart failure with preserved ejection fraction
HFrEF: heart failure with reduced ejection fraction
LAA: left atrial appendage
LAa: left atrial area
LAAV: left atrial appendage emptying velocity
LAT: left atrial thrombus
LVEF: left ventricular ejection fraction
LVDd: left ventricular end-diastolic diameter
NOACs: non-vitamin K antagonist oral anticoagulants
SEC: spontaneous echocardiographic contrast
TEE: transesophageal echocardiography
TIA: transient ischemic attack
TTE: transthoracic echocardiography
VKAs: vitamin-K antagonists.
