# Supplementary material for: Risk factors for left atrial thrombus in younger patients (aged < 65 years) with atrial fibrillation or atrial flutter: Data from the multicenter left atrial thrombus on transesophageal echocardiography (LATTEE) registry
Source: Front Cardiovasc Med. 2022 Oct 12;9:973043. doi: 10.3389/fcvm.2022.973043 (PMC9611536; doi:10.3389/fcvm.2022.973043)
Supplement: Supplementary file 1 [file Data_Sheet_1.docx]

Table S1. Detailed eta squared values for statistically significant quantitative parameters.

| **Variable** | **Patients <65 years**  **LAT(-) vs. LAT(+)**  P value/eta^2^ |
| --- | --- |
| CHA_2_DS_2_VASc score [points] | < 0.001  eta^2^ = 0.03 |
| Haemoglobin [g/dL] | 0.50  eta^2^ = 0.002 |
| eGFR [ml/min/1.73m^2^] | 0.23 |
| LVEF [%] | < 0.001  eta^2^ = 0.05 |
| LA area [cm^2^] | < 0.001  eta^2^ = 0.02 |
| LAA emptying velocity [cm/s] | < 0.001  eta^2^ = 0.09 |
| Abbreviations: eGFR, estimated glomerular filtration rate; LA, left atrial; LAA, left atrial appendage; LVEF, left ventricular ejection fraction/ | |
